# A spatial statistical framework for the parametric study of fiber networks: Application to fibronectin deposition by normal and activated fibroblasts

**DOI:** 10.1017/S2633903X23000247

**Published:** 2023-11-13

**Authors:** Anca-Ioana Grapa, Georgios Efthymiou, Ellen Van Obberghen-Schilling, Laure Blanc-Féraud, Xavier Descombes

**Affiliations:** 1 Université Côte d’Azur, INRIA, CNRS, i3S, France; 2 Université Côte d’Azur, INSERM, CNRS, iBV, France; 3 Université Côte d’Azur, CNRS, INRIA, i3S, France

**Keywords:** Extracellular matrix, fibronectin, graph networks, oncofetal isoforms, statistical parametric maps

## Abstract

Due to the complex architectural diversity of biological networks, there is an increasing need to complement statistical analyses with a qualitative and local description of their spatial properties. One such network is the extracellular matrix (ECM), a biological scaffold for which changes in its spatial organization significantly impact tissue functions in health and disease. Quantifying variations in the fibrillar architecture of major ECM proteins should considerably advance our understanding of the link between tissue structure and function. Inspired by the analysis of functional magnetic resonance imaging (fMRI) images, we propose a novel statistical analysis approach embedded into a machine learning paradigm, to measure and detect local variations of meaningful ECM parameters. We show that parametric maps representing fiber length and pore directionality can be analyzed within the proposed framework to differentiate among various tissue states. The parametric maps are derived from graph-based representations that reflect the network architecture of fibronectin (FN) fibers in a normal, or disease-mimicking in vitro setting. Such tools can potentially lead to a better characterization of dynamic matrix networks within fibrotic tumor microenvironments and contribute to the development of better imaging modalities for monitoring their remodeling and normalization following therapeutic intervention.

## Impact Statement

Quantification of phenotypic variation during tissue development and/or disease progression is essential for the understanding of different pathologies. All organs and tissues contain a non-cellular core component known as the extracellular matrix (ECM), composed of a network of macromolecules whose architecture depends on the pathophysiological state of the tissue. To derive a meaningful comparison of ECM between healthy and diseased tissues, computational frameworks that account for the localization of areas of phenotypic variation are needed. Here we introduce a novel framework for the statistical analysis of parametric maps calculated from graph-based representations of fibers composed of fibronectin, a provisional ECM component that guides ECM organization. Our framework is inspired by the statistical analysis of functional magnetic resonance imaging parametric maps and is embedded in a machine-learning model to compare distinct states of ECM fiber networks, both quantitatively and qualitatively. These methods may be further developed and implemented in ECM profiling of tumor/fibrotic tissue to provide both valuable insight into specific roles of ECM landscapes and their remodeling in disease, and more specific diagnostic, prognostic, and predictive companion biomarkers in the clinic.

## 1. Introduction

During normal development and disease progression, tissues undergo various remodeling processes, which can, in turn, affect their physical characteristics, yielding heterogeneous morphologies. Automated detection and quantification of these phenotypic changes in the tissue landscape are essential for an accurate characterization of a given pathology. Statistical tests that are commonly used for the comparison of two different conditions based on the distributions of morphological properties, are applied at a global scale, and do not account for any explicit spatial information. Here we were interested in exploiting a known spatial statistical approach (historically proposed for functional imaging (fMRI) analysis) and recasting it into a machine learning framework to facilitate the comparison of two tissue conditions. Within the proposed framework relying on statistical parametric mapping (SPM)^(^^1^ ^,^^2^ ^)^, the comparison of various physical tissue characteristics is thus achieved both at a quantitative and qualitative level. It does so by enabling the localization and quantification of local variations of certain morphological properties in the sample that are significantly different and relevant to a given pathology.

SPM is a long-established methodology, specifically developed in fMRI for the detection of significantly activated regions of the brain in a given image sample. Mapping of activated regions is achieved by assessing the probability of random occurrences of activated regions with pixel intensities higher than a given threshold or having a larger spatial extent at lower intensities.

To address the need for taking spatial localization into account when designing frameworks that can discriminate between two different conditions of a given tissue, we recast the SPM paradigm into a data-driven machine learning framework for the detection of significant parametric differences between the two classes. Thus, we train the model on a given population (for example the control case) and we detect local areas in the second population of samples that deviate from this model. In our work, we applied this approach to the characterization of two distinct states of the extracellular matrix (ECM), a non-cellular component of organs and tissues.

The ECM is a biological scaffold with multiple forms and functions. It acts as a biomechanical and structural support ensuring tissue integrity, it relays chemical and physical signals to the residing cells through cell surface receptors and it sequesters growth factors and regulates their bioavailability. The composition and architecture of the ECM is tissue- and organ-specific, and depends on the pathophysiological state of the tissue (i.e., normal vs diseased)^(^^3^ ^)^. For example, while a healthy connective tissue displays a loose meshwork-like ECM, a fibrotic or cancerous stroma is characterized by the presence of dense, aligned ECM fibrils. Thus, the physical and structural traits of the tumor matrix have recently drawn much attention as cancer hallmarks and potential therapeutic targets^(^^4^ ^,^^5^ ^)^. Collagen, the most abundant matrix component, has been extensively investigated in this context and several studies addressing its structural features and their association with cancer progression, metastasis, and treatment have been published^(^^6^ ^–^^8^ ^)^. Collagen deposition, however, depends on the presence of fibronectin (FN), a dimeric glycoprotein that forms a provisional matrix framework into which other ECM components integrate to generate a mature ECM^(^^9^ ^,^^10^ ^)^.

During inflammation, wound healing, or tumor development, the expression of FN is induced and assembled into an insoluble matrix. This FN produced primarily by fibroblasts, corresponds to cellular FN, as opposed to plasma FN. At the molecular level, cellular FN differs from plasma FN by the presence of one or two 90-amino-acid-long alternatively spliced sequences, termed extra domains (EDB and EDA). Extra domain-containing FN displays enhanced assembly, making it the most prevalent FN isoform in diseased tissue. This enhanced FN deposition results in a highly modified ECM architecture with increased fiber density, directionality, and stiffness, that together tune cellular responses and impact tissue homeostasis^(^^11^ ^)^.

Despite the pivotal role of FN in health and disease^(^^12^ ^)^, comprehensive studies of FN fiber features are lacking. In our previous work^(^^11^ ^)^, we set out to develop a robust pipeline of numerical analyses for the extraction of biologically relevant metrics to discriminate among different isoforms of cellular FN from confocal microscopy images of FN matrices. The goal of the present work was to capture and analyze physiologically relevant ECM fibrillar features that discriminate between normal and diseased states. To that end, we generated FN-rich ECM using an *in vitro* model of cell-derived matrices (CDMs) produced by normal fibroblasts, or fibroblasts activated with transforming growth factor beta-1 (TGF- 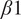 ), a fibrosis-promoting cytokine known to induce a tumor-like state.

Herein we show that the proposed SPM-based machine learning methodology can be used to distinguish between normal and disease-mimicking FN fiber networks. To capture and localize significant differences in fiber properties, we built parametric maps, such as fiber length and pore directionality relying on a graph-based fiber representation that recapitulates the FN fiber networks from confocal microscopy images. In the following sections, we will provide an outline of the proposed methodology applied to SPMs and describe how our proposed machine-learning embedding can yield significant localized parametric variations between different tissue states.

## 2. SPM and Gaussian Random Fields

### 2.1. SPM statistical framework

SPMs are used to evaluate the probability of change in every pixel by using decision tests based on the magnitude of the SPM values (i.e., the peak intensity of a cluster in SPM) as well as the spatial extent of these clusters formed at certain intensity thresholds^(^^1^ ^)^. The value of the pixel intensity reflects a parametric value of interest. Hence, these two-dimensional (2D) maps are constructed to reflect the spatial variation of a measured parameter which is important for discriminating between two given classes with regards to its intensity and area. In this way, clusters of high intensity of an SPM can correspond to a high localized parametric variation, while a large region reflects a spatially extended area of variation.

Our proposed spatial statistical learning framework relies on graph-derived parametric maps to quantify and simultaneously localize statistically significant differences across normal and disease-mimicking FN organizations. Using the pixel intensity of the maps along with the extent of the region area, these differences can be assessed both quantitatively and qualitatively, and detected as anomalies with respect to a Gaussian random field (GRF), corresponding to regions within the maps that cannot be explained by the GRF model learnt from the reference population. Hereafter, we describe the theoretical framework of GRF^(^^13^ ^)^ that enables the statistical analysis of tissue parametric maps.

GRFs, whose marginal distributions are Gaussian vectors 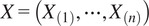 , are characterized by the probability density function:
(1)

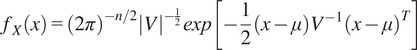

where 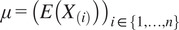 is the expectation and 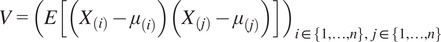 is the covariance matrix.

We consider clusters of pixels as connected components that are formed based on 8-pixel connectivity. Hence, upon image binarization according to a chosen threshold, the pixels (with intensity equal to 1) are grouped together in disjoint components (including single-pixel components) based on similar values of the neighboring eight pixels. It was shown in^(^^13^ ^)^, and later adopted in fMRI-specific studies^(^^1^ ^,^^2^ ^,^^14^ ^)^, that for large thresholds 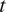 , the clusters are independent and the expectation of the number of clusters at a threshold 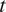 , of an image modeled by a zero-mean, homogeneous Gaussian field of dimension 2, is estimated by the expected Euler characteristic of the excursion set of the GRF:
(2)

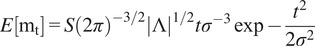

where 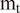 represents the number of clusters at a certain threshold 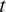 , S is the number of pixels of the image, 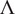 is the covariance matrix of partial derivatives of the GRF, and 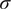 is the standard deviation of the GRF.

Similarly, the mean value of the number of clusters at a threshold 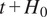 can be written as follows:
(3)

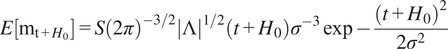

Considering 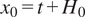 as the intensity peak of a cluster (at threshold 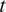 ), one can estimate the probability that a cluster (at a threshold 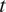 , having an intensity peak higher or equal to 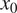 , denoted 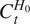 ) belongs to a realization of this GRF, 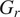 . This probability, as shown in^(^^1^ ^)^, termed 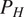 , can be seen as the likelihood of a cluster (formed at threshold 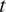 ) of having an intensity peak higher or equal to 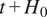 ^(^^1^ ^)^:
(4)

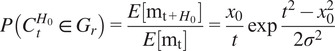

Next, we were interested in the estimation of the probability that a cluster (at a threshold 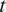 ) belongs to a realization of GRF, depending on its surface (spatial extent - number of pixels). To estimate the number of pixels ( 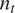 ) of a cluster at a threshold 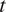 , we use the following equation from^(^^2^ ^)^:
(5)

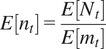

where 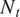 is the number of pixels at of higher intensity than 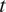 , and 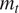 is the number of clusters at the threshold 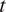 . Since the intensity values follow a normal (zero mean value) distribution, the expectation of 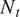 is the following^(^^2^ ^)^:
(6)

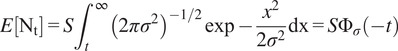

where 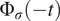 is the complementary cumulative distribution function. It follows, then, based on Equations (2, 5, 6) that one can approximate the mean value of 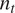 , accordingly:
(7)

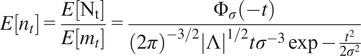

Furthermore, 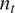 follows an exponential distribution law^(^^15^ ^)^, which is commonly defined by a parameter 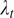 , (the inverse of the mean expected value of the random variable). Consequently:
(8)

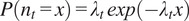

where 
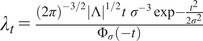


It follows then that the approximation for the probability 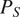 of a given cluster 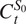 having a spatial extent 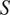 greater than 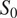 , is given by the following formulation, as shown in^(^^2^ ^)^:
(9)

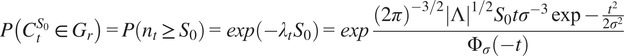

In the following section, we illustrate our proposed approach using a simple scenario of a simulated GRF, in which the model parameters are estimated from a “normal” sample, to detect significant changes in an “abnormal” example.

### 2.2. Test for anomaly detection using synthetic data

**Figure 1.**
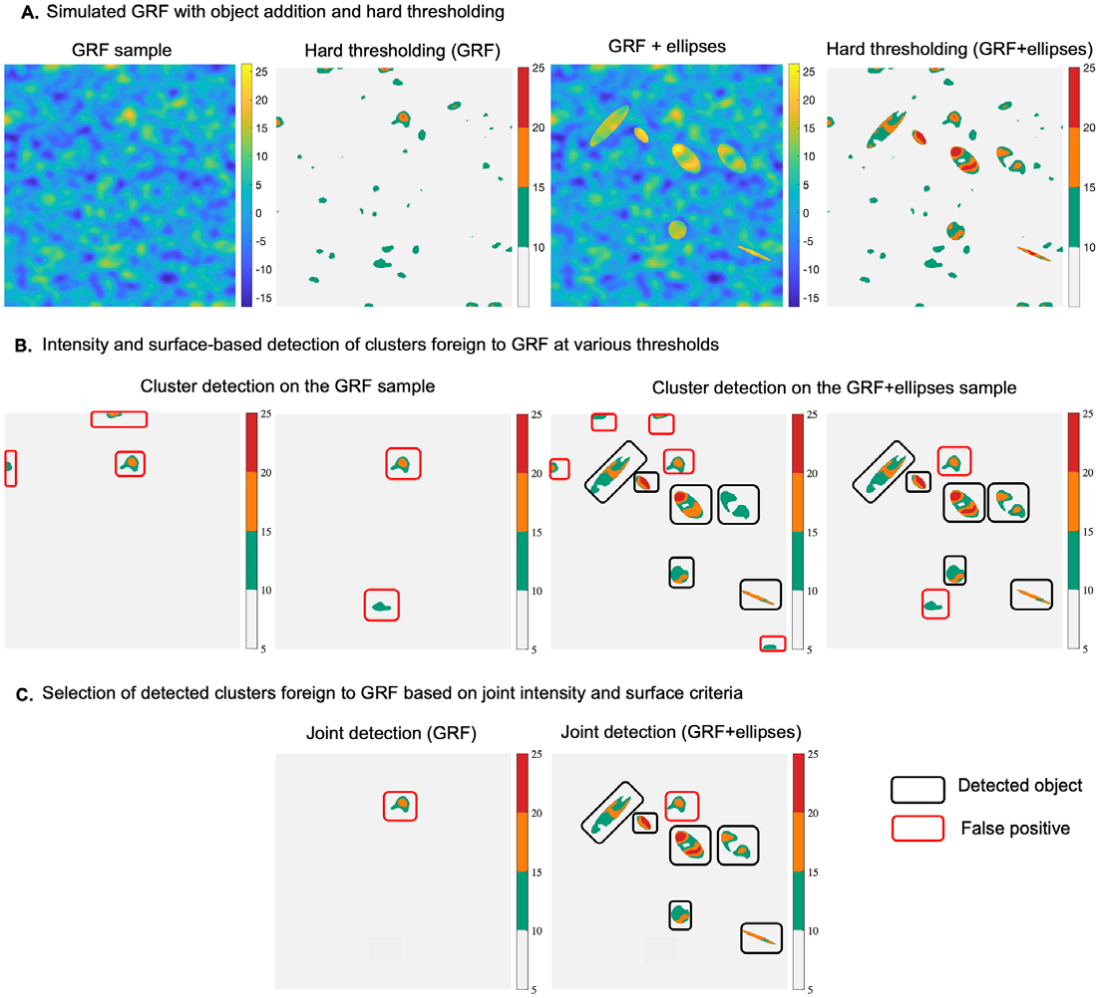
(a) Realization of a GRF of zero-mean (normal case) and addition of 6 different sized ellipses (abnormal case), along with intensity-based hard thresholding for each case, respectively (at a threshold 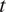 = 10 pixels) (b). Intensity and surface-based detection at pval 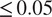 , and at thresholds equal to (10,15,20, see colorbar). GRF sample detection will typically contain false-positive detections while within the abnormal case (GRF+ellipses), all “foreign” objects, that is, ellipses are detected at pval 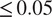 , along with a few false-positives. (c). Clusters that are jointly detected based on the surface and intensity criteria are selected for both normal and abnormal samples.

To better understand the concept of detecting statistical abnormalities in a GRF realization, we considered a simple scenario which includes a synthetic example (normal), representing a simulation of a GRF of zero mean (Figure 1a). To this example we added 6 foreign objects representing ellipses with different surfaces, and intensity levels. The aim was to use our approach to detect these 6 objects within the abnormal sample, showcasing the potential to localize these anomalies at various thresholds, based on the maximum intensity of the different regions detected at each threshold, or their spatial extent (Figure 1b). The null hypothesis is that clusters of pixels computed at different thresholds in the abnormal example belong to a realization of the same GRF as the normal sample. The hypothesis is rejected if either 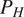 or 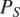 is less or equal than a *p*-value (pval) of 0.05.

Our method learns the GRF model parameters from the reference example, and then uses these parameters to compute the two probabilities of belonging to GRF for each region at various thresholds in the abnormal example. As shown in Figure 1a, b, the current method, compared to a naïve hard thresholding (threshold equal to 10), is better suited to localize the abnormal elements, that is, ellipses, at thresholds equal to (10,15,20). One could opt to jointly consider intensity and surface-based criteria when selecting the detected objects, which in this scenario, would lead to selecting the 6 ellipses together with one false positive (Figure 1c). It is noteworthy that the same false positive is detected on the reference image, which is consistent with the definition of the pval.

### 2.3. Machine-learning embedded in a GRF-based statistical parametric map framework

Providing both a quantitative and a qualitative assessment of the parameter variations is imperative for the study of spatial heterogeneity of fibers in pathological conditions. We were interested in leveraging our proposed framework for the comparison of two distinct conditions, normal and pathological ECM, given parametric maps that reflect various fiber characteristics. To do so, we learnt the GRF model’s parameters from the normal samples and using these parameters, we subsequently determined the probabilities of regions within the pathological conditions belonging to the same GRF, casting the original framework into a machine-learning setting. More specifically, we applied our proposed framework to determine whether fiber length and pore directionality can discriminate between normal and disease-mimicking states. While topographical differences between ECM of healthy and tumor tissue have been described^(^^6^ ^)^, mainly for collagen, no current computational study can, to our knowledge, simultaneously localize and quantify them.

**Figure 2.**
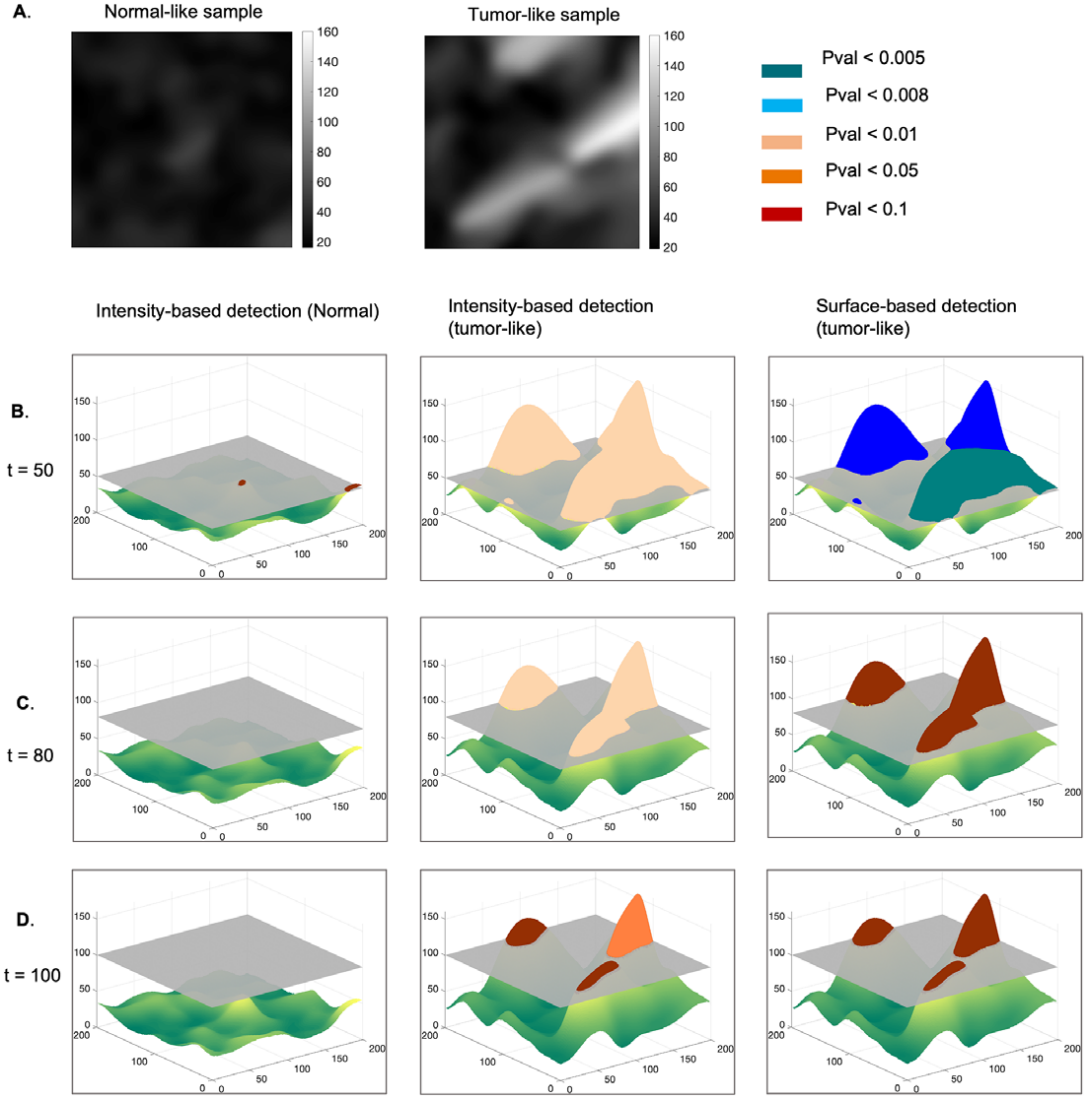
Methodology for statistical detection of foreign regions to a GRF, in an example of a sample representing normal and tumor-like parametric maps. (a) Normal and tumor-like fiber length maps. The normal sample is modeled as a realization of a GRF, and we assume that the tumor-like sample is a realization of the same process. Clusters of regions with an intensity higher than a given threshold, t = 50 (b), t = 80 (c), 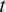 = 100 (d) are found to be statistically different to the GRF, with respect to a pval, depending on the cluster maximum intensity value or their surface.

First, we describe the principle behind the proposed approach (Figure 2), which relies on modeling the normal maps as realizations of a GRF and testing this hypothesis on tumor-like maps^(^^2^ ^)^. We hypothesized that the tumor-like maps are realizations of the GRF learnt from the reference maps and determined a set of probabilities that characterize a degree of belonging to the GRF, for certain contiguous regions (clusters) at various intensity thresholds. In other words, the current statistical analysis identifies those foreign regions with respect to the reference GRF, within both types of maps, under the null hypothesis (i.e., at a given pval)).

In this context, the parametric maps are described by the union of two classes of pixels: those representing a realization of a GRF modeling the normal case, and those that are foreign to the GRF. We expect these foreign elements to occur in regions with very high pixel intensity and/or in larger clusters taken at a specific threshold.

Modeling the parametric maps with GRF is only possible upon gaussianization (i.e., conversion of the empirical distributions into normal distributions) of the GRF marginal distributions. In practice, we only performed the gaussianization of the first-order marginal distribution of the GRF, that is, the image intensity histogram, considering that the parameter maps under study were smooth enough. Therefore, the image intensity histogram was the only distribution to be gaussianized using an approach based on optimal transport^(^^16^ ^,^^17^ ^)^ (Supplementary Figure S1). Thereby, the resulting intensity histogram follows a normal distribution of zero mean with identical variance as the empirical native histogram.

To estimate the likelihood of a certain cluster formed at an intensity threshold 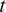 to belong to a GRF, depending on the maximal intensity of this cluster, we relied on the formulations taken from the theory of random fields^(^^13^ ^)^, as previously described. Considering 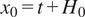 as the intensity peak of a cluster (at intensity threshold 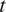 ), one can estimate the probability that a cluster having an intensity peak higher or equal to 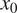 , belongs to a realization of GRF. This probability can be seen as the likelihood of a cluster (taken at threshold 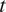 ) of having an intensity peak higher or equal to 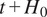 (Equation 4). Furthermore, as previously shown, the approximation for the probability of a given cluster having a spatial extent S greater than 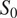 is given by Equation 9. At pval 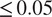 , the clusters of pixels identified at 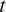 are considered significantly different from the normal GRF model.

In our experiments, we focused on two different FN parametric maps that could potentially discriminate between normal and pathological conditions, fiber length and pore directionality maps, in both reference and disease-mimicking states. We embedded the SPM framework, initially developed to analyze single datasets independently, into a machine learning paradigm. To evaluate the differences between two given groups of maps (e.g., FN in normal state vs disease-like state), we considered one of the groups as the normal realization of the GRF which we divided into a learning set and a smaller test set. The second group was tested for anomalous regions, therefore all the images belonging to this group were considered part of the test set. The proposed method learns the normal GRF model specific parameters, that is, the average value of 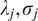 , from the training set. These learnt parameters during the learning phase were subsequently used to compute the two relevant probabilities, 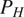 and 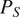 , as described hereafter:

For all the images 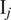 (previously Gaussianized) in the learning set:

• Computation of 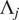 (Equation 2, empirical estimator of the covariance of partial derivatives of 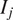 ). If for an image function 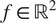 , we consider its gradient vector 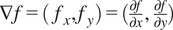 , then 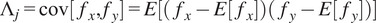 .

• Computation of 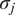 , as the 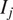 ’s sample standard deviation.

The last step involves storing the average 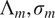 of the learning dataset.

During the test phase, for all the clusters identified at a threshold 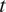 within the test set, 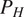 and 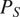 are evaluated using the model’s previously learnt parameters. At pval 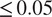 , the clusters are significantly different from the normal GRF model, and can be considered for subsequent analysis (e.g., quantification).

For all the images 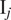 in the test set:

• Gaussianization of each sample image 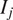 . The result is a new image 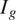 , whose histogram is Gaussian with identical variance to that of 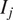 .

• For a given list of thresholds 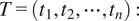


– Binarization of the image 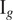 according to the threshold 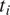


– Once the list of connected components in the binary image resulted from thresholding is achieved, then for every (labeled) connected-component 
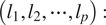


* Evaluation of 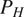 using the learnt model parameter 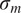 .

* Evaluation of 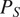 using the learnt model parameters 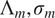 .

## 3. Generation of FN Variant-Specific Fibroblast-Derived Matrices and Induction of a Tumor-Like State

**Figure 3.**
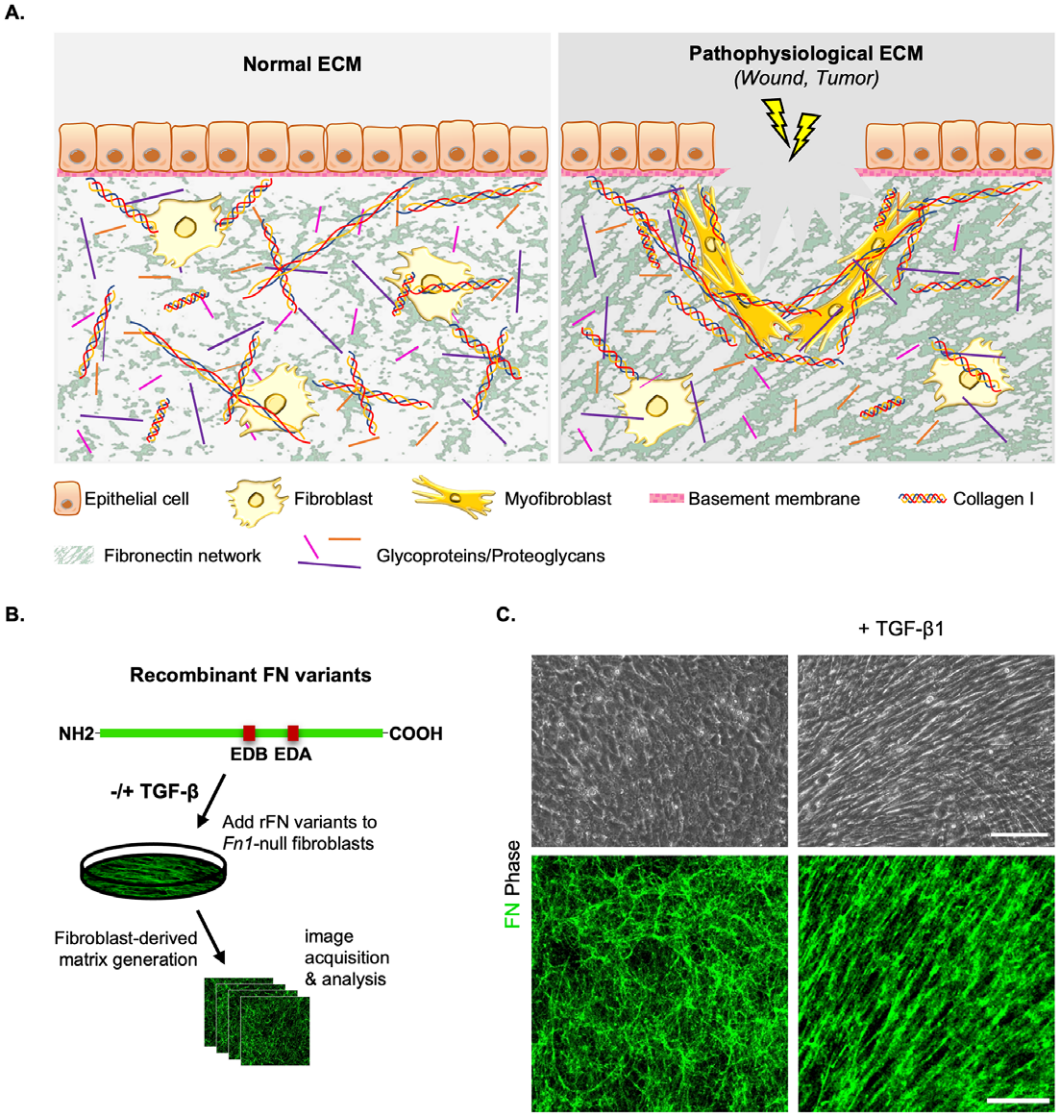
(a) Schematic representation of a simple cuboidal epithelium displaying the different architectures of the underlining ECM in normal (left) and pathological conditions (right). (b) Workflow diagram featuring the linear structure of the purified recombinant FN (rFN) variants and the relative positions of the alternatively spliced Extra Domains, the generation of fibroblast-derived matrices, and image acquisition and analysis. (c) Phase contrast images (top row) of FN-null mouse fibroblasts presented with FN B+A+ variant (15 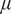 g/ml) in the presence or absence of TGF- 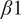 (5 ng/ml) to mimic the changes that take place in the tumor/fibrotic microenvironment. After removal of the cells, matrices were stained with a rabbit-anti-FN polyclonal antibody and visualized with confocal microscopy. Scale bars: phase, 100 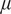 m; IF, 50 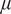 m.

To test our method, we utilized a previously established in vitro system to generate normal and disease-mimicking ECMs by normal mouse fibroblasts. Fibroblasts are the major ECM-producing cells of tissues. In pathological conditions (e.g., tumors), quiescent fibroblasts become activated by environmental cues that induce their phenotypic conversion to “myofibroblasts” with a pro-tumoral phenotype (Figure 3a, top). This process is characterized by the upregulation of cellular FN expression, actin reorganization and increased cell contractility that result in their elongation and the deposition of a highly anisotropic FN-rich ECM^(^^11^ ^)^. In vitro, these changes can be mimicked by treating normal resting fibroblasts with TGF- 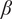 1, a potent cytokine involved in fibroblast activation in the tumor microenvironment^(^^11^ ^)^. For our analyses, FN-rich normal or tumor-like matrices were generated by presenting FN-null mouse embryo fibroblasts with recombinant cFN (prepared as previously described^(^^11^ ^)^), as schematized in Figure 3b. For the induction of a tumor-like phenotype, fibroblasts were incubated with TGF- 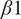 (Figure 3c). Cultures were decellularized after 7 days, and the resulting cell-derived matrices were visualized by immunofluorescence staining and confocal microscopy. Organization of variant-specific FN matrices in normal and activated states was then quantitatively analyzed, as described below.

## 4. Generation of Parametric Maps from Confocal Images of ECM

### 4.1. Fiber detection using Gabor filters and graph extraction

**Figure 4.**
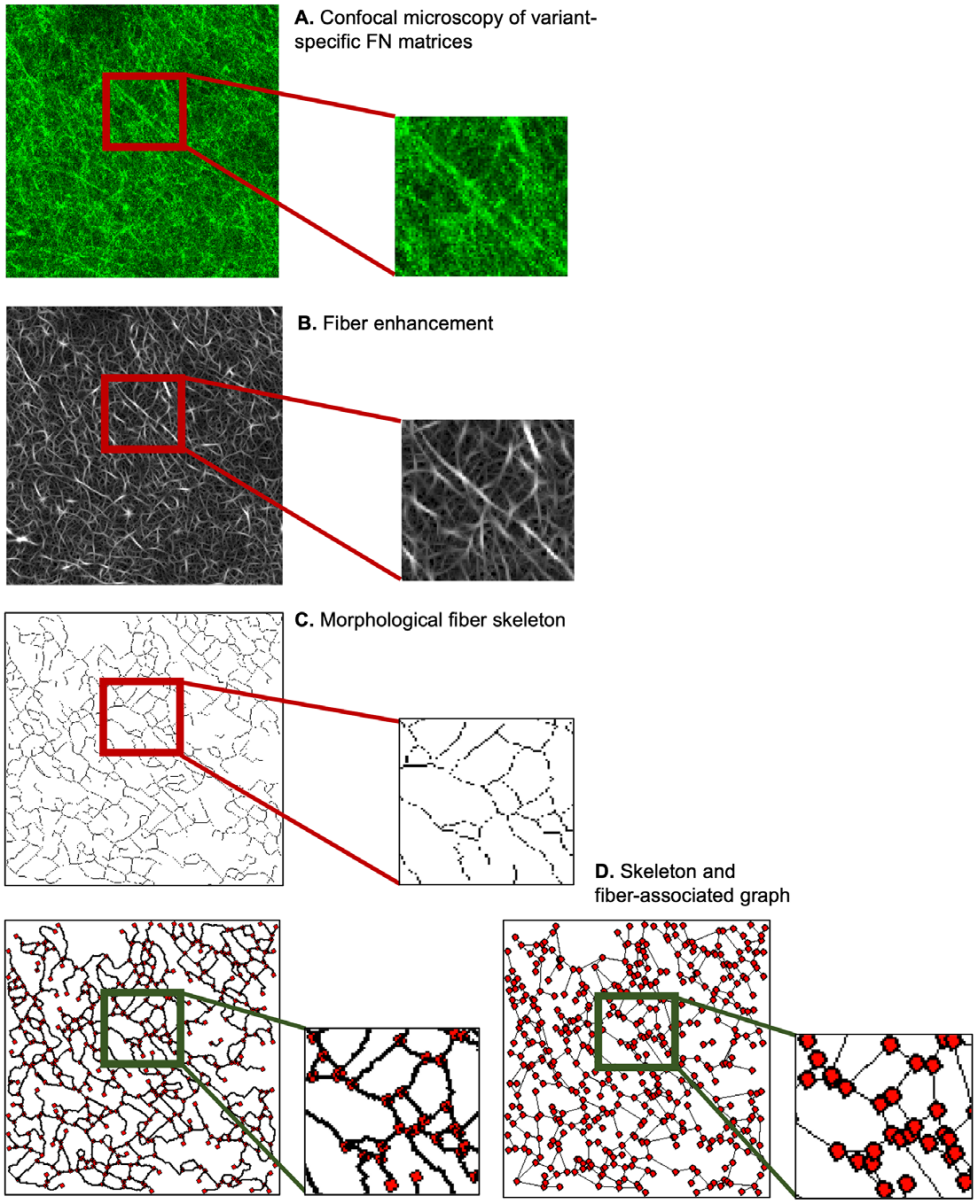
Fiber enhancement and graph-based representation starting from confocal 2D images. (a) Representative region (512x512 pixels) of a sample image (FN B-A+) at a resolution of 0.27 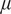 m/pixel. (b) Fiber enhancement with Gabor filters (c) Morphological fiber skeleton extraction (d) Skeleton-based graph (left) and simplified graph representation (right) which is derived from the latter.

To detect and quantify fiber-specific properties of FN networks (Figure 4a), we developed a pipeline that was primarily utilized for the extraction of local topological fiber properties from 2D confocal microscopy images, as described previously^(^^11^ ^)^. Existing ECM analysis methods focusing on collagen exploit alternative fiber detection techniques, such as Fast Fourier Transform bandpass filters^(^^18^ ^)^, ridge detection^(^^19^ ^)^, or fast discrete curvelet transform^(^^20^ ^)^, which is arguably the best suited method to detect curvilinear anisotropic objects, among the previous options. The latter option was used in conjunction with a fiber extraction algorithm. Our method relies on a more flexible detection scheme using Gabor filters, thereby avoiding translation/rotation errors, and unlike other methods, associates graph networks to fiber morphological skeletons, enabling diverse fiber analyses. The current study builds on our previously described fiber enhancement approach^(^^11^ ^)^, for which the key steps are summarized as follows. Fibers in confocal images (Figure 4a) were detected and enhanced using Gabor filters (Supplementary S1) tailored to capture a range of different fiber elements that occur at multiple frequencies and orientations (Figure 4b). Subsequently, we opted for a graph-based framework to construct morphological fiber skeletons (Figure 4c) that would ultimately provide a geometrical characterization of fiber patterns. Further steps for improving fiber representation (e.g., fiber pruning, post-processing fiber reconnection) were implemented as described in previous work^(^^21^ ^)^. Graphs (i.e., collections of nodes connected by edges) are powerful tools for the structural and pattern analysis of objects, which can be utilized for the mathematical study of relations between entities, including fiber-like object detection^(^^22^ ^,^^23^ ^)^.

Within our current analysis, we employ two different graph types to measure fiber-specific properties. First, graphs are used to depict a morphological skeleton representation (Figure 4d, left). Here, the nodes represent either fiber crosslinks (actual fiber junctions or junctions due to the 2D projection of the network onto the image plane) or fiber ends, and the edges capture the fiber length between two given nodes. We previously showed how such a representation can provide a description of distinct local features among four FN variant networks, in a normal state^(^^11^ ^)^.

The second type of graph-based representation introduced in this work (Figure 4d, right) is meant to simplify fiber delineation, as described hereafter. Starting from the skeleton graph, we kept all nodes corresponding to fiber extremities, and connected all pairs of nodes with a straight line, if a fiber had previously been identified. For the sake of simplicity, we refer to fiber length as the length of any straight line connecting a pair of graph nodes.

We note that both representations are useful to extract different local or global fiber properties. The graph-based skeleton fiber delineation faithfully represents (according to a visual assessment performed by a trained biologist) the geometrical and topological properties of the fibers from the 2D confocal images, while the Gabor-specific (e.g., fiber local orientation, thickness) and graph-derived parameters (e.g., fiber length, number of nodes, etc.) are linked to meaningful physical fiber attributes. This biologically relevant representation enabled us to develop here a statistical analysis of the variation of certain fiber parameters for both the normal and a tumor-like state of the FN networks.

### 4.2. Generation of fiber parametric maps from graph-derived fiber representations

**Figure 5.**
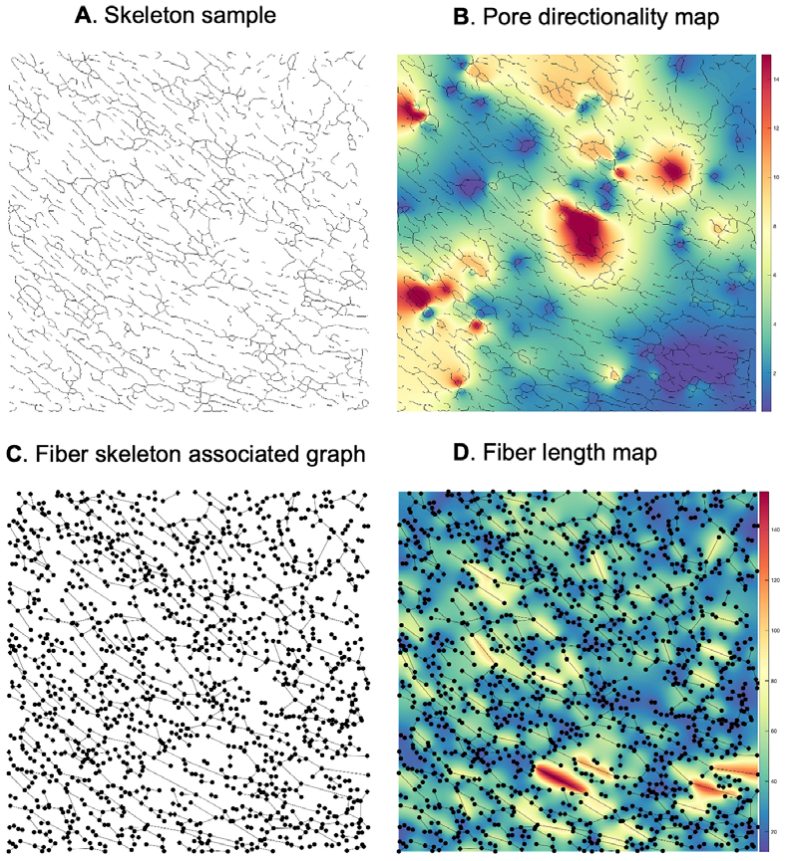
Computation of fiber parametric maps: (a) Starting from the skeleton graph (FN B-A+ disease-like sample, 1024x1024 pixels, 0.27 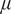 m/pixel), a pore directionality map is derived (b), as the inverse value of the difference between the median pore angle and each individual one. (c) Starting from the fiber skeleton associated graph, a parametric map (fiber length, (d)) associates the fiber length, in pixels, to each corresponding connecting line.

We next sought to develop a statistical framework for differentiating between parametric maps of activated and non-activated FN network configurations, computed from graph-based fiber representations. To create tissue variation maps (Figure 5), we considered different fiber attributes computed from graphs, representing either morphological skeletons (Figure 5a) or simplified graph depictions (Figure 5c). This framework is exemplified on two different types of parametric maps (Figure 5b,d) reflecting discriminative features, namely the individual fiber lengths (i.e., the length of the connecting line between two graph nodes), and the fiber pore (“gap”) directionality (i.e., the inverse value of the absolute difference between the median and individual pore orientation).

To build a fiber length map (Figure 5d), the starting point was the simplified graph-based representation, where nodes depict fiber ends or crosslinks, and fibers are represented by the straight connecting line between these nodes. During the next step, we identified the 2D pixels coordinates that approximate the straight line between the nodes^(^^24^ ^)^ and assigned the length value of the connecting line to each one of the corresponding pixels. The last step for generating dense fiber length maps consists of including the extrapolation of the fiber length values^(^^25^ ^)^ and smoothing of this result with a Gaussian kernel. Concerning the pore directionality parametric maps (Figure 5b), the starting point was the skeleton graph. Pore orientation was computed by first fitting ellipses to each pore and was subsequently obtained by measuring the angle i between the horizontal axis and the major ellipse axis. Pore median value was then subtracted from each i to remove image rotations from the analysis. Finally, the inverse absolute value of the resulting individual score per pore (indicative of the directionality) was assigned to all pixels filling its corresponding surface, and subsequently smoothed out with a Gaussian kernel. High values within the pore directionality maps correspond to those regions in which pores are oriented similarly to the median pore angle, ultimately indicating regions characterized by a predominant pore orientation.

To complement these analyses, we developed a graphical user interface (GUI) for the analysis of a single image/batch displaying fiber networks. Fibers are enhanced using Gabor filters and represented by graphs. Parametric maps, such as fiber length and pore directionality maps can subsequently be derived. The output results can be written into .png image files, while the fiber specific graph/Gabor-derived features are collected in .csv files. The MATLAB source code and sample images for testing can be found on the GitHub platform, at github.com/aigrapa/ECM-fiber-graph.

### 4.3. Test for anomaly detection using fiber network simulations

**Figure 6.**
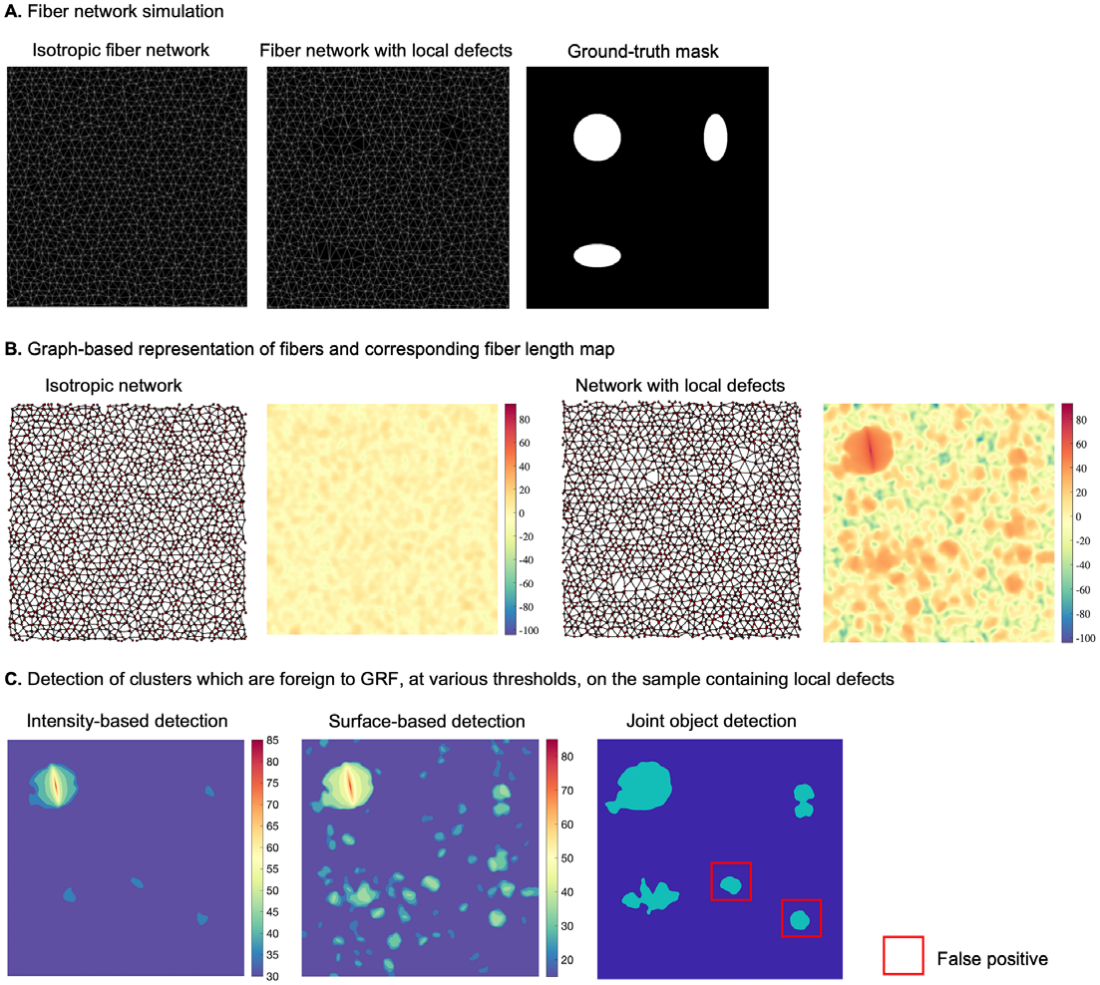
Anomaly detection within parametric maps of simulated fiber networks (a) Simulations of fiber networks (1024x1024 pixels), isotropic (left) and with local defects (center), ground-truth mask (right). (b) Graph-based representations of fiber networks, and corresponding fiber length parametric maps for both samples. (c) Detection of anomalous clusters with respect to the normal GRF model (at pval 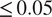 ), at various thresholds, on the parametric map containing defects, for intensity-based (left) and surface-based criteria (center). The regions detected at a threshold of 20, based on a surface-based criterion having a non-null intersection with those detected at a threshold of 35, according to an intensity-based criterion (right).

To simulate fiber networks, we considered a set of scattered point patterns 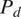 , where 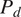 is the set of points 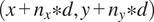 , for 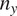 odd, and 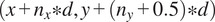 for 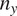 even, 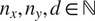 . Random noise was added at the points’ location : 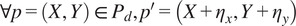 , where 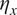 and 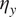 follow a uniform law between 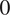 and 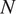 , 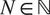 . In this way, we generated two patterns 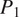 and 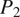 using different values for d, as well as a mask M (for the second scenario), which is an image equal to zero except for specific areas (e.g., ellipses Figure 6a). We consider P as: 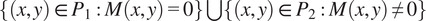 . The fibers were subsequently defined by the edges of the Delaunay graph of P.

The first isotropic fiber network corresponds to a “normal” example ( 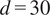 , 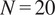 ), while the fiber network with local defects (i.e., fibers are more elongated in the regions containing “defects,” corresponding to the three regions within the mask) is considered here an “abnormal” example ( 
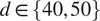
 , 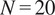 ) (Figure 6a). Fibers were detected as explained in 4.1, and fiber graphs were correspondingly derived for both images (Figure 6b). Starting from the graph-based fiber representation, fiber length parametric maps were generated accordingly, as described in 4.2 (Figure 6b), and subsequently “gaussianised,” as explained in 2.3. We were interested in applying the same principle described in 2.2, in order to detect the three regions of fiber length variation corresponding to the proposed ground-truth mask (Figure 6a). According to this principle, the null hypothesis is that clusters of pixels in the parametric map, computed at different thresholds in the abnormal example, belong to a realization of the same GRF as the normal sample. The method learns the GRF model parameters from the parametric map corresponding to the reference-normal example, and then uses these parameters to compute the two probabilities of belonging to GRF, for each region at various thresholds, in the abnormal sample’s parametric map, using an intensity or surface-based criterion. Different regions were identified at various thresholds, for both intensity and surface-based detection (Figure 6c), at a pval 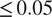 . By only keeping the regions which were detected at a certain threshold (surface-based detection) having a non-null intersection with the clusters detected according to the intensity-based criterion, we were able to accurately detect the three regions of fiber length variation, as well as two additional false positive smaller regions within the parametric map of the fiber network with local defects.

## 5. Results—Statistical Analysis of Fiber Parametric Maps

The graph-based representation of FN networks enabled the subsequent design of a novel framework to perform a spatial statistical analysis of ECM patterns, using graph-derived SPMs. This methodology was applied for a quantitative and qualitative analysis of fiber length and pore directionality differences, across all FN variant networks in normal and tumor-like states. We were thus interested in determining whether the proposed SPM analysis of the selected spatial fiber features could reveal significant variant-specific differences between the FN variant networks in normal (N) and tumor-like (T) states.

**Figure 7.**
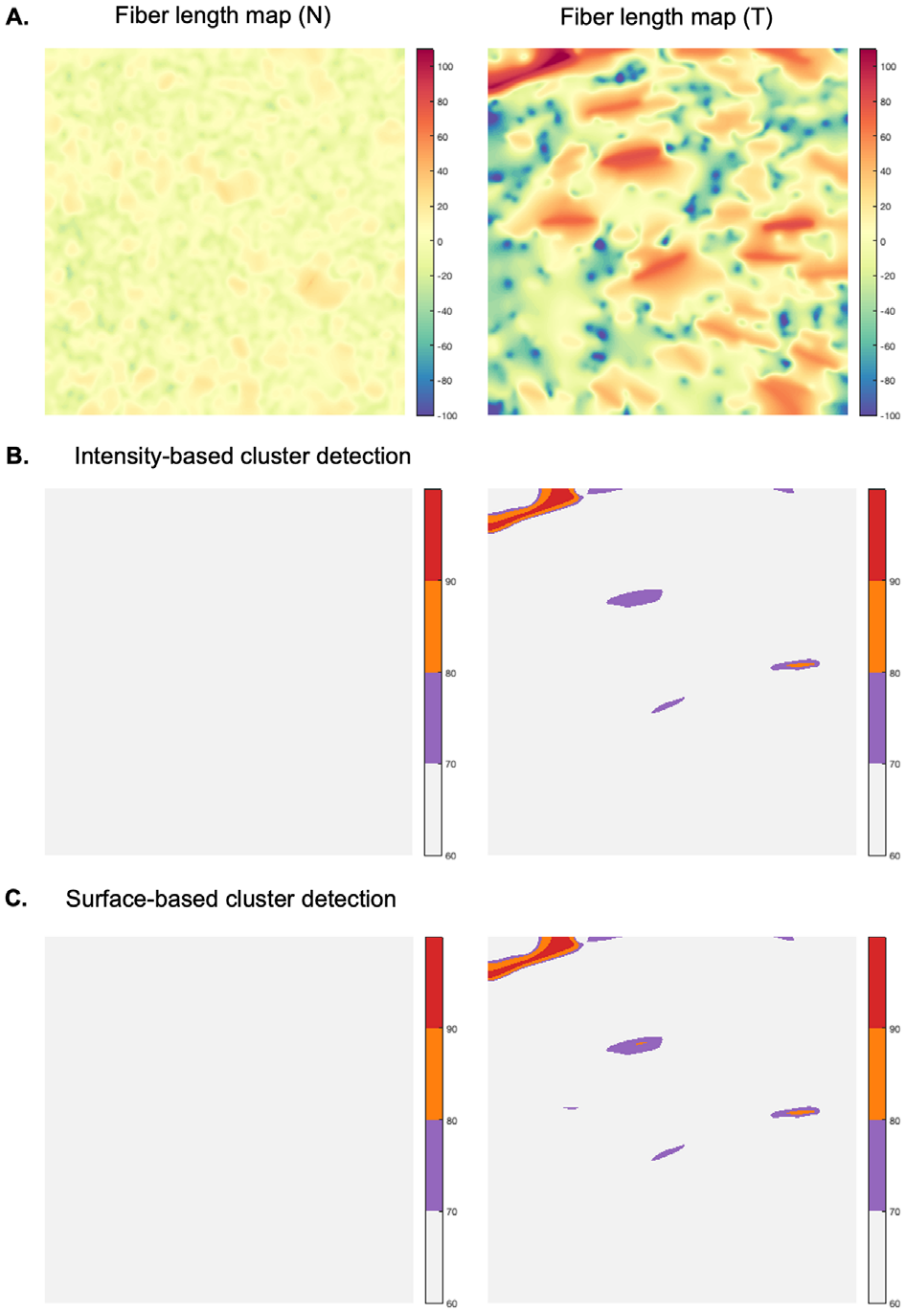
Qualitative analysis—Anomalous cluster detection (with respect to the normal statistical model), applied to two samples of fiber length map (FN B-A+ tumor-like), 1024x1024 pixels, 0.27 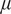 m/pixel (a), (b) and (c) depict the anomalous clusters (pval 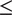 0.05) at various intensity thresholds (70,80,90).

**Figure 8.**
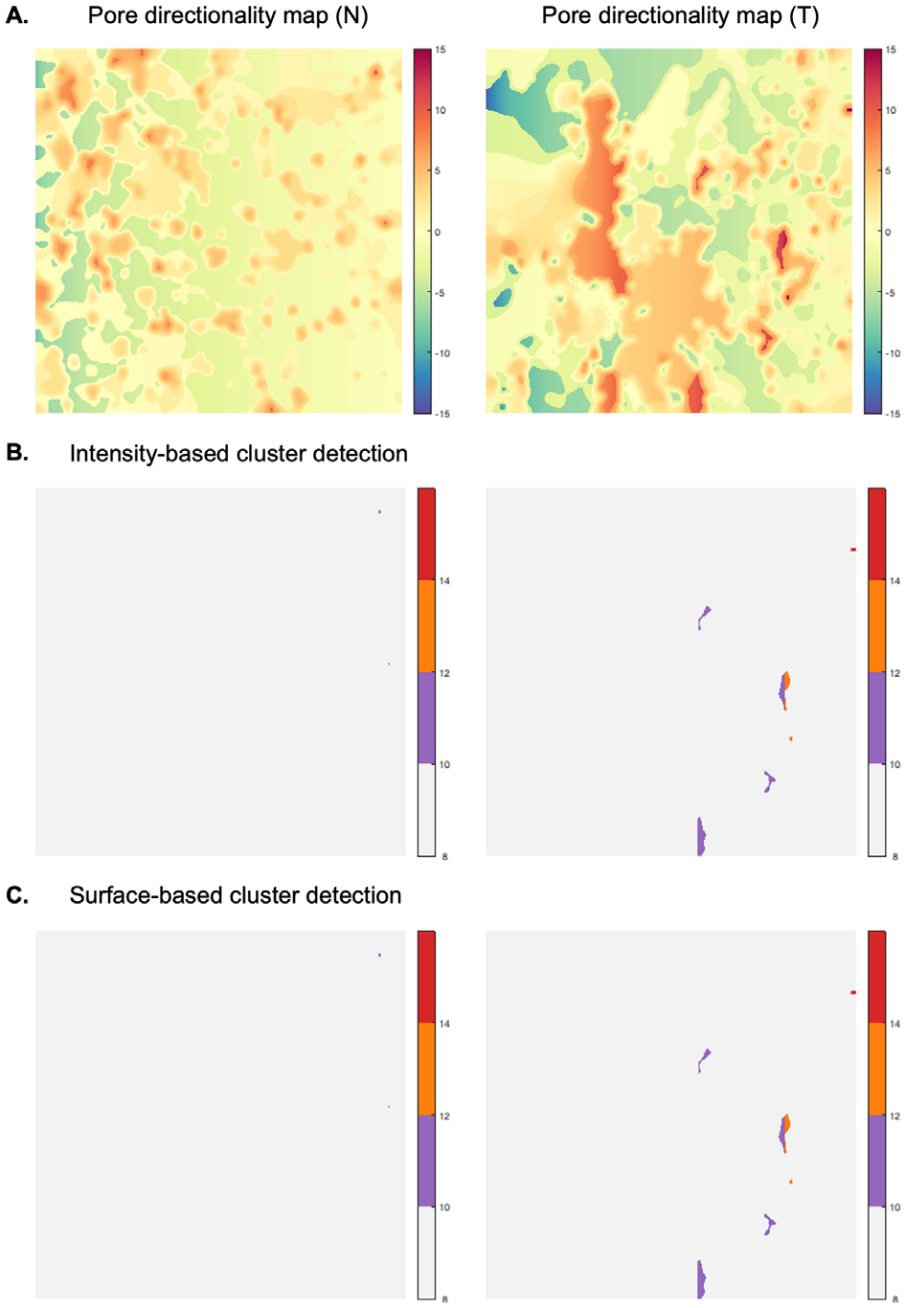
Qualitative analysis—Anomalous cluster detection (with respect to the normal statistical model), applied to two samples of pore directionality map (FN B-A+ tumor-like), 1024x1024 pixels, 0.27 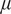 m/pixel (a), (b) and (c) depict the detected clusters (pval 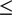 0.05) at various intensity thresholds (10,12,14).

To apply our framework to the available data, we first divided the available sets of confocal images (1024 x 1024 pixels, 0.27 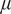 m/pixel; 70 images/variant for normal FN (N) and 65 images/variant for tumor-mimicking FN networks) as follows. For comparison of (N) vs (T) FN networks, we considered 50 (N) samples as the learning dataset, 20 (N) as a test set for normal, and 65 (T) as a test set for disease-like networks. In all scenarios, a cluster is considered significantly different from the normal GRF model at pval 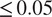 . Anomalies in fiber length (Figure 7a) detected using either intensity or surface-based criteria (Figure 7b,c) and pore directionality maps (Figure 8a–c) were detected at a few intensity thresholds (e.g., 70,80,90) and (10,12,14), respectively (Figures 7, 8). Thus, using our approach, differences in fiber length and pore directionality could be localized in regions formed at different intensity thresholds. This property is very useful for obtaining a qualitative analysis of parametric maps, where clusters of pixels that are statistically different from a normal model can be localized.

For the quantitative analysis of tissue parametric variations, we set out to determine significant differences between FN variant networks through the average number of identified foreign clusters, as well as the average cluster area per image. It is noteworthy that the group for which anomalous clusters were found at superior thresholds, had higher parametric values than in the normal model. For example, if significantly different regions occur in the tumor-mimicking matrices compared to the normal ones, with respect to fiber length, then fibers are statistically more elongated within former networks than normal counterparts. As shown in Figures 7, 8, we found both fiber length and pore directionality to be significantly different for pairwise comparisons of normal and tumor-like FN variant networks. Essentially, the latter type of FN architecture relative to normal ECM, is represented by statistically longer fibers (Table 1) with a more pronounced pore directionality (Table 2). The increased length of FN fibers is consistent with the elongated phenotype of the TGF- 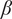 1-treated fibroblasts that assemble them. The statistically significant increase in pore directionality of tumor-like matrices compared to normal FN matrices is in agreement with published reports^(^^26^ ^)^ of higher FN alignment in cell-derived matrices from cancer-associated fibroblasts and in tumor tissue. Detailed results, including the average area and number of identified anomalous clusters, at multiple thresholds, for four FN variants, are presented in Supplementary Tables S1–S8.

**Table 1.** Quantitative analysis for detection of differences in fiber length—Anomalous cluster quantification (with respect to the normal statistical model, at pval 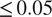 ), for the comparison of normal (N) and tumor-like (T) FN (1024x1024 pixels, or 276.48 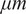 x 276.48 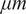 ).

FN variant	Average number of clusters for (N) vs (T) FN
B-A-	0.43 (surface)
B+A-	0.88 (intensity); 0.92 (surface)
B-A+	0.72 (intensity); 0.78 (surface)
B+A+	0.94 (intensity); 0.94 (surface)

*Note.* The average number of significant clusters per test database for each variant is shown here, for either surface or intensity criteria, if higher than for the normal model, at any selected threshold). Average number of clusters detected in fiber length maps, for normal vs tumor-mimicking FN, for one of the test thresholds (70,80,90).

**Table 2.** Quantitative analysis for detection of differences in pore directionality—Anomalous cluster quantification (with respect to the normal statistical model, at pval 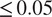 ), for the comparison of normal (N) and tumor-like (T) FN ( 1024x 1024 pixels, or 276.48 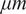 x 276.48 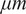 ).

FN variant	Average number of clusters for (N) vs (T) FN
B-A-	0.12 (intensity)
B+A-	0.22 (surface)
B-A+	0.14 (intensity); 0.34 (surface)
B+A+	—

*Note.* The average number of significant clusters per test database for each variant is shown here, for either surface or intensity criteria, if higher than for the normal model, at any selected threshold). Average number of clusters detected in pore directionality maps, for normal vs tumor-mimicking FN, for one of the test thresholds (10,12,14). ‘—‘ is recorded if no significant detection was present.

## 6. Discussion and Conclusion

The proposed methodology was designed to quantify the differences in terms of spatial organization between normal and disease-like architectures of variant-specific FN matrices generated *in vitro.* We have previously been able to discriminate the matrix patterns of four alternatively spliced FN variants deposited by cultured fibroblasts using different learning approaches and relying on graph-based feature analysis. The pipeline which includes steps for fiber detection and representation^(^^11^ ^)^, and generation of parametric fiber maps is available with a MATLAB GUI: both graph and Gabor filter-based fiber features can be extracted from different images representing fiber networks (e.g., ECM-specific proteins) for downstream analysis. In principle, the steps required for the characterization of ECM (i.e., fiber detection and representation using graphs) in cell-derived matrices generated by cells of different origin remain the same. However, in the case of tissue samples, the ECM is more complex and heterogeneous, and further pre-processing steps may be needed to filter structures that are normally found in tissues (e.g., blood vessels). The number and type of additional steps, however, is dependent on the type of organ/tissue, the pathology under evaluation, and the staining procedure.

Here, we developed an SPM framework for the quantitative and qualitative analysis of fibers, capable of simultaneously detecting and measuring variations of specific ECM features within two different tissue conditions. Importantly, our approach can be evaluated on parametric maps at different thresholds, producing results that are more reliable and statistically relevant (by providing a pval) than a simple hard thresholding of the parametric maps. Our framework was tested using two relevant fiber features, fiber length and pore directionality, whose parametric maps revealed significant differences between normal and disease-mimicking states. However, parametric maps can be extended to include other fiber or pore-specific parameters (e.g., fiber density, width, length, orientation, waviness, and straightness), which could be useful for differentiating among various biological networks in normal and pathological states.

Computational analyses of ECM structures can provide essential information about their role in shaping the cellular microenvironment topology in health and during disease progression. Prognostic ECM-specific signatures have already been inferred in cancer-related studies, and in diseases with prominent fibrosis^(^^27^ ^–^^29^ ^)^. There is also a growing interest in the integration of cell and ECM analyses in a spatially resolved manner to further understand the interactions between cells and their matrix microenvironment^(^^30^ ^)^. Indeed, the present work proposing a versatile pipeline for the analysis of ECM produced by cultured fibroblasts, is being extended to studies of ECM organization in human tumor tissue and aims to integrate the phenotypes of cellular components. Hence, a combined local analysis of parametric maps and metrics describing the organization/morphology of adjacent cells (e.g., tumor, immune, vascular cells) will potentially help elucidate the complex interplay between cellular and non-cellular components of the tumor microenvironment.

## 7. Materials and Methods

### 7.1. Materials and FN preparations

Recombinant human TGF- 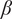 1 was from R 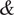 D Systems Inc. (Minneapolis, MN, USA). All other chemicals and reagents were purchased from Sigma Aldrich (St Louis, MO, USA) unless otherwise stated. Purified recombinant FN variants were produced as previously described^(^^11^ ^)^.

### 7.2. Cells and culture conditions

*Fn1 -/-* mouse kidney fibroblasts were generated and cultured as previously described^(^^11^ ^)^. For experiments, FN was depleted from fetal calf serum using gelatin sepharose-4B columns (GE Healthcare, Uppsala, Sweden), and the culture medium was supplemented with Penicillin-Streptomycin 100 U/ml and, where indicated, TGF- 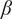 1 (5 ng/ml). Absence of Mycoplasma sp. contamination was routinely verified by PCR as described elsewhere^(^^31^ ^)^.

### 7.3. Generation of fibroblast-derived matrices, immunofluorescence staining and microscopy

Fibroblast-derived matrices were generated as described previously. For FN immunostaining, primary antibody (rabbit polyclonal anti-FN) was from Merck-Millipore (Darmstadt, Germany). Fluorescently-labeled (Alexa Fluor 488-conjugated) secondary antibody was from Thermo Fisher Scientific (Waltham, Massachusetts). After staining, the coverslips were mounted in ProLong Gold antifade reagent (Thermo Fischer Scientific). Confocal imaging was performed on a Zeiss LSM710 confocal system equipped with a 10X/0.45 NA objective. For visual representation, image treatment was performed using Fiji^(^^32^ ^)^.

## Supporting information

Grapa et al. supplementary material Grapa et al. supplementary material

## Data Availability

Sample image data and code can be found on the GitHub platform: github.com/aigrapa/ECM-fiber-graph.

## Supplementary material

The supplementary material for this article can be found at http://doi.org/10.1017/S2633903X23000247.
