# Supplementary material for: A spatial statistical framework for the parametric study of fiber networks: Application to fibronectin deposition by normal and activated fibroblasts
Source: Biol Imaging. 2023 Nov 13;3:e25. doi: 10.1017/S2633903X23000247 (PMC10951922; doi:10.1017/S2633903X23000247)
Supplement: Grapa et al. supplementary material [file S2633903X23000247sup001.pdf]

Supplementary Material

S1. Graph-based representation on top of Gabor filters

We defined a set of Gabor kernels g_k, characterized by an exponential term which provides the shape of a bivariate gaussian kernel multiplied by a cosine function which describes its oscillations in space:

$$g_k \ = exp\,[-\frac{1}{2}v^t(\Sigma_{\theta_i}^{-1})v]cos\left(\frac{2\pi x\theta_i}{\lambda_j}+\phi\right)(1)$$

where we denote by $v=(x,y)^t$, the 2D coordinate vector, indicating pixels localization in a bidimendional Cartesian coordinate system.

To capture the various geometrical properties of the FN fibers using Gabor filters, we have constructed a set of Gabor kernels g_k, $k\ \in\{1,2,\dots 60\}$, defined by the following parameters:

- Fiber orientation is represented by θ_i, $\theta_i=\frac{i\pi}{20}$ where $i=\{0,1,2,\dots 19\}$.
- Fiber thickness is denoted as λ_j, $j=\{1,2,3\}$ and corresponds to the wavelength (in pixels) of the cosine term, the values of which are equal to $\frac{\lambda_j}{2}$ and vary between 3 and 5 pixels. Therefore, the thinnest fibers are detected when λ_j= 6 pixels, medium thickness fibers correspond to λ_j= 8 pixels, while the thickest are characterized by λ_j= 10 pixels.
- For accurate localization of fibers, the phase of the cosine function, ϕ, is set to {0}.
- The spatial support of the kernels is given by $\sigma_x=5$ pixels and $\sigma_y=3$ pixels, indicating an anisotropic filter that is appropriate for fiber detection.

At any given location within the filtered image sample, the Gabor kernel that returns the highest coefficient modulus was retained. Once fibers were enhanced using Gabor filters, we extracted their morphological skeleton, using different morphological operations (e.g. binarization with hysteresis, morphological thinning, etc.). The graph-based representation of the fiber skeletons was subsequently derived, using a toolbox that produces a network graph assigned to a morphological skeleton (1) . To reconstruct the missing fibers, we used Dijkstra's weighted shortest path algorithm, which finds for each node (i.e. fiber end), the shortest path to other extremities or nodes of the skeleton graph, which are within a given distance to the considered node. Among all possible paths, we chose the minimal one, relying on the intensity from the Gabor filtered image. Additionally, the reconnection of the fibers

was made using the angle of the Gabor maximum response as a guideline, which resulted in the reconnection of the fibers within a predefined cone sector around this local fiber orientation.

S2. 1D Optimal Transport

The approach that was considered for map gaussianization was based on the optimal transport framework (2). More specifically, the problem of "converting" the empirical intensity distribution of the fiber length map into a normal distribution with the same variance, can be approached by performing the 1D optimal transport between the two distributions. Briefly, for a map I with an empirical mean and variance of intensity, we consider a second image J, whose intensity pixels follow a normal distribution (Figure S1). The 1D optimal transport problem will determine how to optimally permute the pixels indices in J, to "recreate" the image I. Since the intensity of the pixels in the permuted version of J does not change, the result will resemble I, but will have the histogram of J (3).

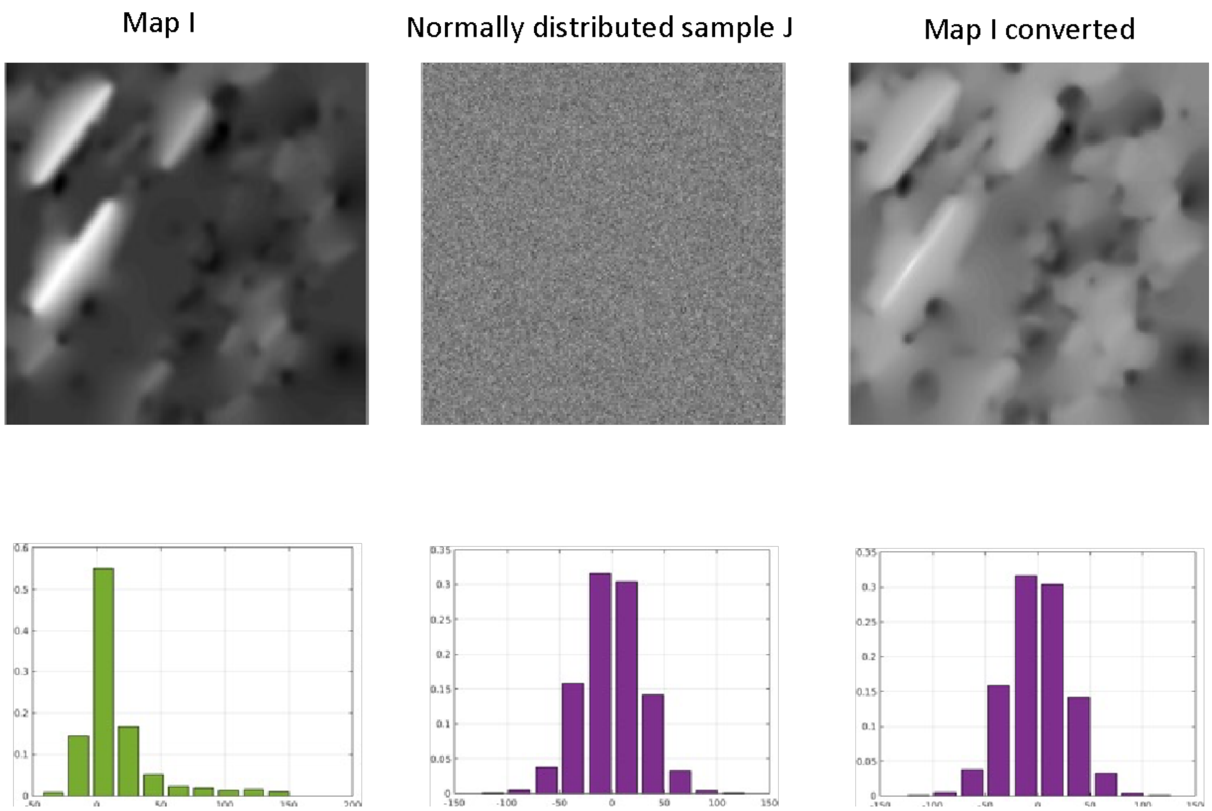

S1 Fig. *Gaussianization through optimal transport: Top row illustrates an example of a fiber length parametric map (Map I), a generated image J with normally distributed intensity around 0, and equal variance to that of Map I, and, finally, the converted map I with a corresponding normally distributed intensity. Bottom row shows the corresponding intensity histograms*

S3. Quantitative results (area and number) of detected clusters foreign to a GRF upon comparison of normal and tumor-like FN variants

For each of the four FN variants (B-A-, B+A-, B-A+, B+A+), the normal and tumor-like form are compared using the proposed method as described in the main paper, Section 5 (Results – statistical analysis of fiber parametric maps). Steps are reiterated here: we first divided the available sets of confocal images (1024 x 1024 pixels, 0.27 μm/pixel; 70 images/variant for normal FN (N) and 65 images/variant for tumor-mimicking FN networks) as follows. For comparison of (N) vs (T) FN networks, we considered 50 (N) samples as the learning dataset, 20 (N) as a test set for normal, and 65 (T) as a test set for disease-like networks. Comparisons between FN variants with respect to fiber length maps are shown in Table S 1, Table S 2, Table S 3, Table S 4, and pore directionality in Table S 5, Table S 6, Table S 7, Table S 8.

Table S 1. Foreign cluster detection (with respect to the normal statistical model), applied for the comparison of B-A- (N) and B-A- (T) FN (276.48μm x 276.48 μm) fiber length maps. Clusters are detected according to intensity and surface criteria, at pval ≤ 0.05, for various thresholds 70,80,90. The average number (count) and area of detected clusters are recorded, where the results corresponding to the normal model are shown in the first half of the table.

	B-A- vs B-A-	Thresholds				B-A- vs B-A-	Thresholds		
		70	80	90			70	80	90
Intensity	Count	0.10	0.05	0.05	Intensity	Count	0.11	0.02	0.00
	Area	432.50	68.00	16.00		Area	2066.5	1069.00	0.00
Surface	Count	0.00	0.00	0.00	Surface	Count	0.43	0.29	0.15
	Area	0.00	0.00	0.00		Area	772.11	273.00	95.30

	B+A- vs B+A-	Thresholds				B+A- vs B+A-	Thresholds		
		70	80	90			70	80	90
Intensity	Count	0.00	0.00	0.00	Intensity	Count	0.88	0.51	0.26
	Area	0.00	0.00	0.00		Area	3608.82	2194.45	1165.65
Surface	Count	0.15	0.10	0.10	Surface	Count	0.92	0.89	0.78
	Area	220.67	44.50	7.00		Area	3127.58	1315.53	506.22

Table S 2. Foreign cluster detection (with respect to the normal statistical model), applied for the comparison of B+A- (N) and B+A- (T) FN (276.48μm x276.48 μm) fiber length maps. Clusters are detected according to intensity and surface criteria, at pval ≤ 0.05, for various thresholds 70,80,90. The average number (count) and area of detected clusters are recorded, where the results corresponding to the normal model are shown in the first half of the table.

	B-A+ vs B-A+	Thresholds				B-A+ vs B-A+	Thresholds		
		70	80	90			70	80	90
Intensity	Count	0.00	0.00	0.00	Intensity	Count	0.72	0.40	0.23
	Area	0.00	0.00	0.00		Area	6038.85	6265.31	6553.13
Surface	Count	0.15	0.10	0.10	Surface	Count	0.78	0.54	0.42
	Area	38.00	5.00	1.50		Area	5337.84	4563.03	3591.33

Table S 3 . Foreign cluster detection (with respect to the normal statistical model), applied for the comparison of B-A+ (N) and B-A+ (T) FN (276.48μm x 276.48 μm) fiber length maps. Clusters are detected according to intensity and surface criteria, at pval ≤ 0.05, for various thresholds 70,80,90. The average number (count) and area of detected clusters are recorded, where the results corresponding to the normal model are shown in the first half of the table.

	B+A+ vs B+A+	Thresholds				B+A+ vs B+A+	Thresholds		
		70	80	90			70	80	90
Intensity	Count	0.00	0.00	0.00	Intensity	Count	0.94	0.49	0.29
	Area	0.00	0.00	0.00		Area	3609.21	2509.34	1511.63
Surface	Count	0.05	0.00	0.00	Surface	Count	0.94	0.74	0.71
	Area	17.00	0.00	0.00		Area	3298.38	1574.79	675.59

Table S 4. Foreign cluster detection (with respect to the normal statistical model), applied for the comparison of B+A+ (N) and B+A+ (T) FN (276.48 μm x 276.48 μm) fiber length maps. Clusters are detected according to intensity and surface criteria, at pval ≤ 0.05, for various thresholds 70,80,90. The average number (count) and area of detected clusters are recorded, where the results corresponding to the normal model are shown in the first half of the table.

	B-A- vs B-A-	Thresholds				B-A- vs B-A-	Thresholds		
		10	12	14			10	12	14
Intensity	Count	0.05	0.00	0.00	Intensity	Count	0.12	0.03	0.00
	Area	1041.00	0.00	0.00		Area	1216.63	429.50	0.00

Surface	Count	0.25	0.20	0.00	Surface	Count	0.31	0.20	0.06
	Area	370.20	37.00	0.00		Area	480.10	99.15	37.25

Table S 5. Foreign cluster detection (with respect to the normal statistical model), applied for the comparison of B-A- (N) and B-A- (T) FN (276.48 μm x 276.48 μm) pore directionality maps. Clusters are detected according to intensity and surface criteria, at pval ≤ 0.05, for various thresholds 10,12,14. The average number (count) and area of detected clusters are recorded, where the results corresponding to the normal model are shown in the first half of the table.

	B+A- vs B+A-	Thresholds				B+A- vs B+A-	Thresholds		
		10	12	14			10	12	14
Intensity	Count	0.10	0.05	0.00	Intensity	Count	0.35	0.05	0.00
	Area	947.50	292.00	0.00		Area	979.43	558.33	0.00
Surface	Count	0.30	0.05	0.00	Surface	Count	0.42	0.22	0.15
	Area	400.83	278.00	0.00		Area	482.00	187.79	39.30

Table S 6. Foreign cluster detection (with respect to the normal statistical model), applied for the comparison of B+A- (N) and B+A- (T) FN (276.48 μm x 276.48 μm) pore directionality maps. Clusters are detected according to intensity and surface criteria, at pval ≤ 0.05, for various thresholds 10,12,14. The average number (count) and area of detected clusters are recorded, where the results corresponding to the normal model are shown in the first half of the table.

Table S 7. Foreign cluster detection (with respect to the normal statistical model), applied for the comparison of B-A+ (N) and B-A+ (T) FN (276.48 μm x 276.48 μm) pore directionality maps. Clusters are detected according to intensity and surface criteria, at pval ≤ 0.05, for various thresholds 10,12,14. The average number (count) and area of detected clusters are recorded, where the results corresponding to the normal model are shown in the first half of the table.

	B-A+ vs B-A+	Thresholds				B-A+ vs B-A+	Thresholds		
		10	12	14			10	12	14
Intensity	Count	0.15	0.00	0.00	Intensity	Count	0.54	0.14	0.02
	Area	561.67	0.00	0.00		Area	1169.49	513.44	238.00
Surface	Count	0.15	0.05	0.00	Surface	Count	0.52	0.34	0.15
	Area	478.33	132.00	0.00		Area	651.68	162.27	43.50

	B+A+ vs B+A+	Thresholds				B+A+ vs B+A+	Thresholds		
		10	12	14			10	12	14
Intensity	Count	0.15	0.05	0.05	Intensity	Count	0.11	0.03	0.00
	Area	3933.33	2804.00	469.00		Area	1034.43	368.00	0.00
Surface	Count	0.35	0.20	0.05	Surface	Count	0.18	0.09	0.05
	Area	1565.43	771.50	89.00		Area	633.42	170.33	35.33

Table S 8. Foreign cluster detection (with respect to the normal statistical model), applied for the comparison of B+A+ (N) and B+A+ (T) FN (276.48 μm x 276.48 μm) pore directionality maps. Clusters are detected according to intensity and surface criteria, at pval ≤ 0.05*, for various thresholds 10,12,14. The average number (count) and area of detected clusters are recorded, where the results corresponding to the normal model are shown in the first half of the table.*

References

1. Kollmannsberger P, Kerschnitzki M, Repp F, Wagermaier W, Weinkamer R, Fratzl P. The small world of osteocytes: Connectomics of the lacuno-canalicular network in bone. New Journal of Physics. 2017 Jul 1;19(7).

2. Peyré G, Cuturi M. Computational Optimal Transport. arXiv:180300567 [stat] [Internet]. 2020 Mar 18 [cited 2021 Oct 12]; Available from: http://arxiv.org/abs/1803.00567.

3. Peyré G. The Numerical Tours of Signal Processing - Advanced Computational Signal and Image Processing. [Internet]. IEEE Computing in Science and Engineering; 2011. Available from: https://hal.archives-ouvertes.fr/hal-00519521/document.
